# A global cline in a colour polymorphism suggests a limited contribution of gene flow towards the recovery of a heavily exploited marine mammal

**DOI:** 10.1098/rsos.181227

**Published:** 2018-10-17

**Authors:** J. I. Hoffman, E. Bauer, A. J. Paijmans, E. Humble, L. M. Beckmann, C. Kubetschek, F. Christaller, N. Kröcker, B. Fuchs, A. Moreras, Y. D. Shihlomule, M. N. Bester, A. C. Cleary, P. J. N. De Bruyn, J. Forcada, M. E. Goebel, S. D. Goldsworthy, C. Guinet, A. R. Hoelzel, C. Lydersen, K. M. Kovacs, A. Lowther

**Affiliations:** 1 Department of Animal Behaviour, Bielefeld University, 33501 Bielefeld, Germany; 2 British Antarctic Survey, High Cross, Madingley Road, Cambridge CB3 OET, UK; 3 Department of Zoology and Entomology, Mammal Research Institute, University of Pretoria, Private Bag X20, Hatfield 0028, South Africa; 4 Norwegian Polar Institute, Fram Centre, 9296 Tromsø, Norway; 5 Antarctic Ecosystem Research Division, Southwest Fisheries Science Center, National Marine Fisheries, National Oceanographic and Atmospheric Administration, 8901 La Jolla Shores Drive, La Jolla, CA 92037, USA; 6 South Australian Research and Development Institute, 2 Hamra Avenue, West Beach, South Australia 5024, Australia; 7 Centre d'Etudes Biologiques de Chizé (CEBC), CNRS and Université de La Rochelle - UMR 7372, 79360 Villiers en Bois, France; 8 Department of Biosciences, Durham University, South Road, Durham DH1 3LE, UK

**Keywords:** colour polymorphism, melanocortin 1 receptor gene, population structure, fur seal, pinniped

## Abstract

Evaluating how populations are connected by migration is important for understanding species resilience because gene flow can facilitate recovery from demographic declines. We therefore investigated the extent to which migration may have contributed to the global recovery of the Antarctic fur seal (*Arctocephalus gazella*), a circumpolar distributed marine mammal that was brought to the brink of extinction by the sealing industry in the eighteenth and nineteenth centuries. It is widely believed that animals emigrating from South Georgia, where a relict population escaped sealing, contributed to the re-establishment of formerly occupied breeding colonies across the geographical range of the species. To investigate this, we interrogated a genetic polymorphism (S291F) in the melanocortin 1 receptor gene, which is responsible for a cream-coloured phenotype that is relatively abundant at South Georgia and which appears to have recently spread to localities as far afield as Marion Island in the sub-Antarctic Indian Ocean. By sequencing a short region of this gene in 1492 pups from eight breeding colonies, we showed that S291F frequency rapidly declines with increasing geographical distance from South Georgia, consistent with locally restricted gene flow from South Georgia mainly to the South Shetland Islands and Bouvetøya. The S291F allele was not detected farther afield, suggesting that although emigrants from South Georgia may have been locally important, they are unlikely to have played a major role in the recovery of geographically more distant populations.

## 1. Introduction

Evaluating the extent to which natural populations are connected by gene flow is important for understanding how species may respond to anthropogenic exploitation [1]. Classical studies have quantified migration rates using permanent physical tags that allow individuals to be tracked over time and space [2], while more recently the development of genetic markers for many species has facilitated the widespread adoption of population genetic approaches such as assignment testing [3,4], which are capable of distinguishing immigrants from locally born individuals. However, the power of these genetic approaches depends on the number of markers that can be deployed as well as on the strength and pattern of population structure [5,6]. An alternative is therefore to exploit naturally occurring but discrete phenotypic variants, such as colour morphs, to infer migration patterns.

**Figure 1.**
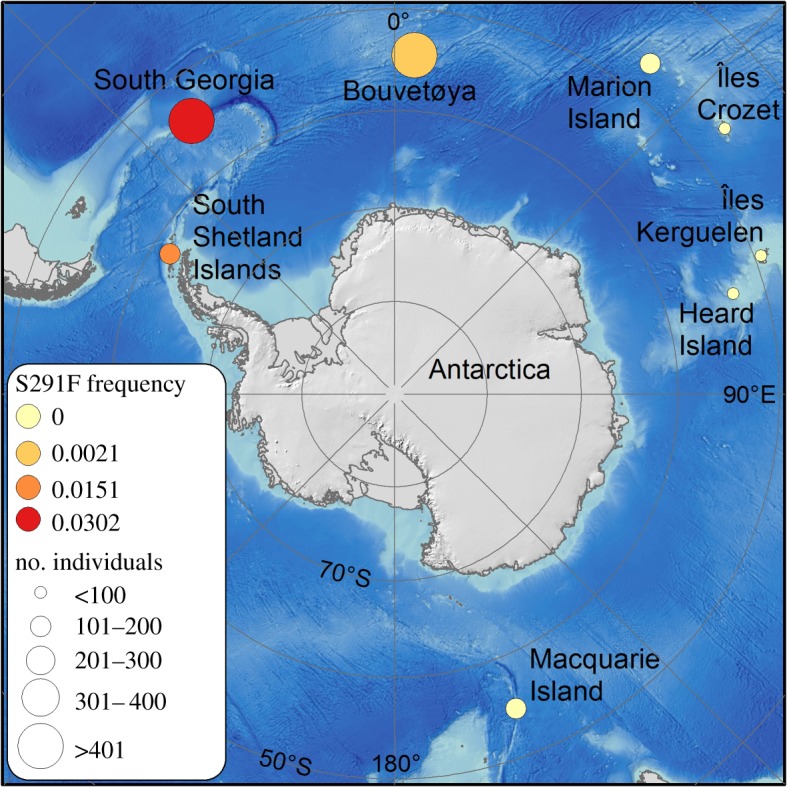
Map showing geographical variation in S291F frequency in Antarctic fur seals. Circle size is proportional to the number of samples sequenced from each of eight different populations spanning the geographical range of the species. S291F frequency is denoted on a colour scale ranging from red (the highest frequency at South Georgia) to cream (the allele was not detected in the sample).

An interesting test case is provided by the Antarctic fur seal (*Arctocephalus gazella*), a pinniped species that breeds on sub-Antarctic islands (figure 1) with 97% of the contemporary population concentrated around South Georgia in the South Atlantic [7]. Females exhibit strong natal philopatry [8] and both sexes are also highly faithful to breeding territories held in previous years [9], yet sightings of this species as far afield as Gough Island in the South Atlantic [10], Brazil [11] and Australia [12] indicate the potential for long-distance dispersal. Like many other pinnipeds, Antarctic fur seals were subjected to extreme exploitation for their skins during the eighteenth and nineteenth centuries, with over a million seals taken from South Georgia alone [13]. By the twentieth century, the species was considered virtually extinct [14] although remnant populations may have survived on remote islands off the northwest of South Georgia [15] as well as in the South Shetland Islands [16] and probably also at Bouvetøya [17–19]. Although Antarctic fur seal numbers showed little sign of recovery until the 1930s [20], within just a few decades the species had re-occupied all of its former breeding sites and the worldwide population is now thought to number around four to six million animals (IUCN Red List, http://www.iucnredlist.org).

Exactly how this species staged a global recovery remains an open question. However, the population of fur seals at South Georgia grew rapidly in the 1960s and 1970s and had already reached around 1.5 million animals by the early 1990s [21]. Consequently, several authors have speculated that emigrants from this expanding population may have played an important role in the re-establishment and subsequent growth of formerly occupied colonies across the species range [22,23]. Empirical studies using genetic markers have provided mixed support for this hypothesis. In particular, a mitochondrial study [19] uncovered weak global population structuring and identified three genetically distinctive regions: a western region comprising the populations of South Georgia, the South Shetland Islands, Bouvetøya and Marion Island, an eastern region comprising Îles Kerguelen and Macquarie Island and an intermediate region containing Îles Crozet and Heard Island (figure 1). Similar patterns have also been reported based on nuclear markers [16,24], although genetic differences between populations need not necessarily preclude ongoing migration, which has indeed been documented between South Georgia and the nearby South Shetlands [16]. Consequently, further studies are needed to evaluate how migration may have contributed towards the recovery of these severely depleted populations [25].

**Figure 2.**
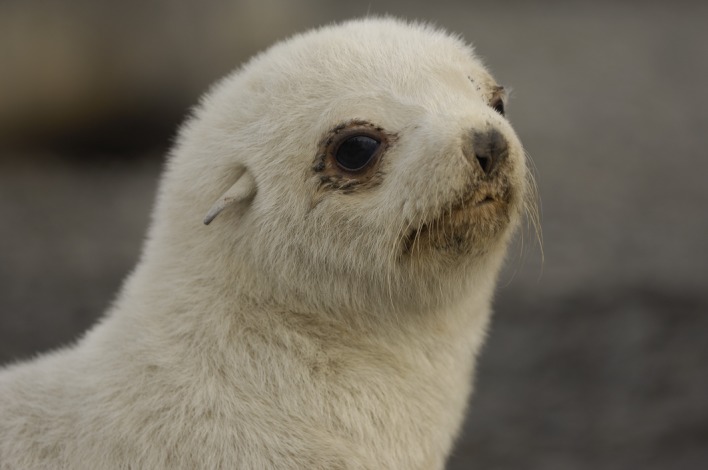
Phaeomelanic Antarctic fur seal pup. Photograph credit: Oliver Krüger.

An intriguing avenue of enquiry is provided by recent observations of hypo-pigmented Antarctic fur seals (figure 2) at a number of sub-Antarctic islands [25,26]. In contrast to wild-type individuals, which have dark brown fur, hypo-pigmented animals have a distinctive cream-coloured (phaeomelanic) phenotype, which is the result of reduced melanin production [27]. Until recently, hypo-pigmented fur seals had only been observed at South Georgia, where their relatively high frequency (one in 600–1400 individuals) is thought to have resulted from a strong historical bottleneck [15,28]. However, phaeomelanic adults have now been sighted at the nearby South Shetland Islands [29–31] as well as at Bouvetøya [32] and Marion Island [25,26], where the first confirmed birth of a hypo-pigmented pup outside of the Scotia Arc was also recently reported [25]. These observations have been interpreted as providing evidence that individuals from South Georgia (or other populations in the Scotia Arc) emigrated to Marion Island carrying with them the allele responsible for hypo-pigmentation [25,26].

The development of a draft Antarctic fur seal genome assembly [24,33] recently allowed the genetic basis of hypo-pigmentation to be elucidated in animals from South Georgia [34]. The melanocortin 1 receptor gene (*MC1R*), which plays a key role in the regulation of pigment production, was sequenced in 70 wild-type and 26 hypo-pigmented pups. This led to the identification of a non-synonymous mutation that results in the substitution of serine with phenylalanine at position 291 in the amino acid sequence, which is an evolutionarily highly conserved structural domain. All of the phaeomelanic animals were found to be homozygous for the allele coding for phenylalanine (S291F), suggesting that a recessive loss-of-function mutation is responsible for cream coat coloration.

Here, we sequenced a short section of the *MC1R* containing the S291F substitution in a large sample of pups representing all of the main Antarctic fur seal breeding localities from across the global distribution of the species. This approach allowed us to evaluate whether gene flow from South Georgia could explain recent sightings of hypo-pigmented individuals beyond the Scotia Arc. As the S291F allele is thought to have originated in South Georgia and cream-coloured animals are considered to be relatively rare at Bouvetøya and Marion Island, we hypothesized that restricted gene flow would result in a decline in S291F frequency with increasing geographical distance from South Georgia.

## 2. Methods

### 2.1. Sample collection

Skin samples were collected from a total of 1492 Antarctic fur seal pups selected at random from within eight different breeding colonies (table 1). Owing to the rarity of phaeomelanic animals, all of the sampled individuals had the wild-type (i.e. dark) phenotype. The seals were captured and restrained on land using standard methodology [35]. Skin samples were taken from the interdigital margin of the foreflipper using piglet ear notching pliers [36] and stored at –20°C in 20% dimethyl sulfoxide saturated with sodium chloride.

**Table 1.** Frequencies of the wild-type (C) and S291F allele (T) in eight Antarctic fur seal populations assigned to three geographical regions as defined by Wynen *et al*. [19]. Corresponding 95% binomial confidence intervals (CIs) are given in parentheses. Three phaeomelanic animals that were specifically targeted during field surveys at Bouvetøya and Marion Island (see Methods) are not included in the table.

region	population	no. individuals	no. C	no. T	S291F frequency (95% CI)
western	South Georgia	496	962	30	0.0302 (0.0205–0.0429)
	Livingstone Island, South Shetlands	199	392	6	0.0151 (0.0056–0.0325)
	Bouvetøya	467	932	2	0.0021 (0.0003–0.0077)
	Marion Island	141	282	0	0 (0–0.0130)
	**total**	1303	2568	38	0.0146 (0.0103–0.0200)
intermediate	Îles Crozet	15	30	0	0 (0–0.1157)
	Heard Island	21	42	0	0 (0–0.0841)
	**total**	36	72	0	0 (0–0.0499)
eastern	Îles Kerguelen	46	92	0	0 (0–0.0393)
	Macquarie Island	107	214	0	0 (0–0.0171)
	**total**	153	306	0	0 (0–0.0120)
grand total		1492	2946	38	

Although a recent study [34] found a clear link between S291F allele homozygosity and hypo-pigmentation at South Georgia, samples from phaeomelanic animals from other breeding colonies were not available at the time. We therefore analysed samples collected from two phaeomelanic animals specifically targeted during field surveys at Bouvetøya, as well as a single phaeomelanic yearling at Marion Island, which is the subject of a detailed account by De Bruyn *et al*. [26].

### 2.2. Sequence acquisition and analysis

Total genomic DNA was extracted using an adapted phenol-chloroform protocol [37]. We then sequenced a 537 base pair (bp) region of the *MC1R* coding region using the primers 5'-ctggagatgggtgcttcttc-3′ and 5'-tctttgtagccatgctggtg-3′ as described in detail by Peters *et al*. [34]. Briefly, purified PCR products were sequenced in both directions using the Applied Biosystems BigDye Terminator v. 3.1 Cycle Sequencing Kit (Thermo Fisher Scientific: Waltham, MA, USA) and analysed on an ABI 3730xl capillary sequencer. The laboratory work was performed at Bielefeld University. Consensus sequences were then generated using ChromasPro v. 1.3.4 and aligned manually within BioEdit v. 5.0.6. The resulting alignment was used to quantify the frequencies of the wild-type and mutant (S291F) allele in each population. Heterozygous sites were identified as those with two peaks of roughly equal intensity but around half the intensity of a homozygote.

### 2.3. Statistical analyses

Binomial 95% confidence intervals corresponding to point S291F frequency estimates were calculated using the *binom.confint* function in the R package *binom*. Fisher's exact tests were then used to analyse pairwise differences in S291F frequency among the eight populations and among the three regions defined by Wynen *et al*. [19]. The resulting *p*-values were corrected for the table-wide false discovery rate (FDR) using the approach of Benjamini & Hochberg [38]. Finally, we tested for a clinal pattern by constructing a generalized linear model (GLM) in which S291F frequency was expressed as a two-vector response variable (number of mutant alleles, number of wild-type alleles) and modelled using a binomial error structure. Geographical distances among the populations were calculated as the shortest routes between each island avoiding land using the geodesic measurement tool in Esri ArcGIS v. 10.6. Geographical distance from South Georgia (in kilometres) was then fitted as a predictor variable and an *F*-test was implemented to determine statistical significance. All data analyses were conducted in R v. 3.3.2.

## 3. Results

We first tested whether S291F homozygosity is responsible for cream coat coloration outside of South Georgia by analysing two phaeomelanic fur seals from Bouvetøya and one from Marion Island. All three of these animals were S291F homozygotes, suggesting that the substitution identified at South Georgia is also responsible for hypo-pigmentation farther afield. Considerable variation was found in S291F frequency among the populations (figure 1 and table 1). South Georgia had the highest overall frequency (0.0302, 95% CI = 0.0205–0.0429) followed by the South Shetland Islands (0.0151, 95% CI = 0.0056–0.0325) and Bouvetøya (0.0021, 95% CI = 0.0003–0.0077). The S291F allele was not detected in any of the other populations, although sample sizes tended to be smaller for the more distant colonies resulting in comparatively large confidence intervals. S291F frequency differed significantly between five of the populations (table 2) as well as between the western and eastern regions (table 3) after FDR correction (pairwise Fisher's exact tests, *p* < 0.05). Moreover, a binomial GLM uncovered a highly significant negative association between S291F frequency and distance from South Georgia (*F*_1,7_ = 44.72, *p* < 0.0001).

**Table 2.** Pairwise Fisher's exact tests at the population level. Odds ratios are given above the diagonal and corresponding *p*-values after table-wide FDR correction are given below the diagonal.

	South Georgia	Livingstone Island, South Shetlands	Bouvetøya	Marion Island	Îles Crozet	Heard Island	Îles Kerguelen	Macquarie Island
South Georgia	—	0.4910	0.0689	0	0	0	0	0
Livingstone Island, South Shetlands	0.1347	—	7.1207	0	0	0	0	0
Bouvetøya	<0.0001	0.0108	—	0	0	0	0	0
Marion Island	0.0011	0.0445	1	—	0	0	0	0
Îles Crozet	1	1	1	1	—	0	0	0
Heard Island	0.6297	1	1	1	1	—	0	0
Îles Kerguelen	0.1028	0.5995	1	1	1	1	—	0
Macquarie Island	0.0057	0.0963	1	1	1	1	1	—

**Table 3.** Pairwise Fisher's exact tests at the regional level. Odds ratios are given above the diagonal and corresponding *p*-values after table-wide FDR correction are given below the diagonal.

	western region	intermediate region	eastern region
western region	—	0	0
intermediate region	0.6247	—	0
eastern region	0.0287	1	—

## 4. Discussion

We analysed a genetic polymorphism responsible for cream coat coloration in Antarctic fur seals to test the hypothesis that emigrants from the expanding South Georgia population contributed towards the recovery of breeding colonies across the species' former geographical range. S291F frequency declined steeply with increasing geographical distance from South Georgia suggesting that, although gene flow occurs on a local scale, it is unlikely that emigrants from South Georgia played a major role in the recovery of geographically more distant populations.

### 4.1. Genetic basis of hypo-pigmentation

It has been suggested that the mutation responsible for hypo-pigmentation in Antarctic fur seals arose at South Georgia, where it drifted to high frequency due to a strong historical bottleneck [15] and later spread to other localities such as the South Shetlands, Bouvetøya and Marion Island [25]. In line with this and consistent with previous results based on a larger sample size of individuals from South Georgia [34], we found that phaeomelanic individuals from Bouvetøya and Marion Island were homozygous for the S291F allele. Although we were only able to sample three phaeomelanic individuals from these localities, our results are suggestive of a conserved genetic mechanism as opposed to multiple mutations in the *MC1R* gene having arisen independently in different populations. Consequently, it seems reasonable to assume that spatial variation in the frequency of hypo-pigmented animals will be a function of underlying differences in S291F frequency.

### 4.2. S291f allele frequency at South Georgia

Previously, Bonner [28] used direct counts of individuals sighted ashore to estimate the frequency of hypo-pigmented individuals at South Georgia. He produced three successive estimates ranging from one in 1400 in 1956 (*n* = 2809 sighted seals) through one in 800 in 1957 (*n* = 4968 sighted seals) to one in 600 in 1962 (*n* = 5400 sighted seals). In this study, the observed frequency of the S291F substitution in South Georgia was 0.0302, which corresponds to an expected frequency of S291F homozygotes of one in 1096 (95% CI = 1:154–1:2380) assuming Hardy–Weinberg equilibrium. Consequently, our genetic estimate of the frequency of phaeomelanic individuals at South Georgia falls squarely within the range of Bonner's [28] estimates, suggesting that our methodology is appropriate for quantifying variation on a global scale.

### 4.3. Spatial variation in S291F frequency

Hypo-pigmented fur seals have previously been sighted at South Georgia [28], the South Shetland Islands [29], Bouvetøya [32] and more recently at Marion Island [25,26]. However, data on the frequencies of hypo-pigmented animals at locations other than South Georgia are currently lacking. We found that S291F allele frequency declined steeply with increasing geographical distance from South Georgia. Specifically, the S291F allele was estimated to be around half as abundant at the South Shetlands in comparison to South Georgia and around 15 times less abundant at Bouvetøya. Although our population-level estimates had rather large 95% confidence intervals owing to the rarity of the S291F mutation, our data are overall indicative of an isolation-by-distance pattern.

Farther afield at Marion Island in the sub-Antarctic Indian Ocean, two independent studies have reported sightings of hypo-pigmented Antarctic fur seals [25,26], including a recent description of the birth of a phaeomelanic pup [25]. This led Wege *et al*. [25] to conclude that the allele responsible for hypo-pigmentation has become ‘entrenched’ in the Marion Island population. Our data are consistent with this notion, as the phaeomelanic pup from Marion Island was homozygous for the S291F allele, confirming that the causative mutation is indeed present in this population. However, the S291F allele was not detected in a random sample of pups from Marion Island, suggesting that it cannot be very common. This makes sense given that the S291F allele is already rather rare at Bouvetøya, which is over a thousand kilometres closer to South Georgia.

Moving even further away from South Georgia, we also failed to detect the S291F allele in any of the populations belonging to the intermediate and eastern regions defined by Wynen *et al*. [19]. Our power to detect the allele at these locations is relatively low due to modest sample sizes (ranging from 15 to 107 individuals), which reflect the difficulty of collecting samples from these extremely remote and inaccessible locations. However, significant differences in S291F frequency were found after pooling allele counts by region. This suggests that our results are not purely due to differences in sample size but rather reflect genuine differences in S291F frequency across the species' geographical range.

Our findings complement and build upon previous population genetic studies of Antarctic fur seals [16,19,24]. On a broad geographical scale, the discovery of significant differences in S291F frequency between the western and eastern regions is concordant with Wynen *et al*. [19]. On a finer geographical scale, the pattern of declining S291F frequency moving away from South Georgia within the western region is also consistent with a recent nuclear study reporting genetic differences between South Georgia, the South Shetland Islands and Bouvetøya [24]. Finally, another recent study that focused exclusively on South Georgia and the South Shetlands also found genetic differences between these two populations, but could furthermore show that several pups born at the South Shetlands had recent immigrant ancestries from South Georgia [16]. This is consistent with our having found the second highest S291F frequency at the South Shetlands and supports the suggestion of Wege *et al*. [25] that sightings of hypo-pigmented individuals may provide a marker of ongoing migration [25].

Finally, although locally restricted migration provides a parsimonious explanation for the pattern of decreasing S291F frequency with increasing geographical distance from South Georgia, we cannot discount the possible involvement of non-neutral processes. For example, if hypo-pigmented seals were more prone to predation by top predators such as leopard seals (*Hydrurga leptonyx*) or killer whales (*Orcinus orca*), then geographical variation in phaeomelanism could potentially be a reflection of underlying differences in predation pressure. However, this seems unlikely at least for leopard seals, which are actually more common in the western Antarctic [39,40]. Moreover, comprehensive census data from South Georgia suggest that hypo-pigmented and wild-type animals do not differ in their survival probabilities [34].

## 5. Conclusion

Our study of a heavily exploited circumpolar distributed marine mammal uncovered a global cline in the frequency of a colour polymorphism. This is consistent with previous observational data on hypo-pigmented animals from several locations [25,26,28,29,32] as well as with genetic studies reporting both population structure [19,24] and local migration [16]. Our study therefore contributes to a growing consensus that relict fur seal populations probably survived sealing at multiple locations, at least some of which appear to be connected by ongoing gene flow.

## Supplementary Material

R-code for “A global cline in a colour polymorphism suggests a limited contribution of gene flow towards the recovery of a heavily exploited marine mammal.”

## Data accessibility

The raw data (allele counts) analysed in this paper are provided in table 1. The code used to analyse them are available as an R markdown file, which is provided in electronic supplementary material, S1.
