# Supplementary material for: A global cline in a colour polymorphism suggests a limited contribution of gene flow towards the recovery of a heavily exploited marine mammal
Source: R Soc Open Sci. 2018 Oct 17;5(10):181227. doi: 10.1098/rsos.181227 (PMC6227926; doi:10.1098/rsos.181227)
Supplement: R-code for “A global cline in a colour polymorphism suggests a limited contribution of gene flow towards the recovery of a heavily exploited marine mammal.” [file rsos181227supp1.html]

R-code for A global cline in a colour polymorphism suggests a limited contribution of gene flow towards the recovery of a heavily exploited marine mammal.


# R-code for "A global cline in a colour polymorphism suggests a limited contribution of gene flow towards the recovery of a heavily exploited marine mammal."

#### *J. I. Hoffman, E. Bauer, A.J. Paijmans, E. Humble, L. M. Beckmann, C. Kubetschek, F. Christaller, N. Kröcker, B. Fuchs, A. Moreras, Y. D. Shihlomule, M. N. Bester, A. C. Cleary, P. J. N. De Bruyn, J. Forcada, M.E. Goebel5, S. D. Goldsworthy, C. Guinet, A. R. Hoelzel, C. Lydersen, K. M. Kovacs & A. Lowther*

This document provides the code for the analysis in our paper. The sequence of code follows the sequence of analysis in the *Results* section of the paper.

**Prerequisites:**

- For running the code you need the following libraries and the raw data files `Blondies_no_hybs_dups.csv` and `distance_SG.csv` saved in a `data/raw` directory.

```
library(dplyr)
options(scipen = 999)
library(tidyr)
library(data.table)
library(binom)
library(forcats)
source("R/martin.R")
library(extrafont)
library(ggplot2)
library(knitr)
library(kableExtra)
```

## Loading and summarising the data

```
# Read in allele data wrangled in 1_identify_hybs_dubs.R
# Count the number of blonde and wildtype alleles
# Summarise allele frequencies

data <- read.csv("data/processed/Blondies_no_hybs_dups.csv") %>%
  mutate(No.blonde.alleles = case_when(pos872 == "C" ~ 0,
                                       pos872 == "T" ~ 2,
                                       pos872 == "t" ~ 1)) %>%
  mutate(No.wildtype.alleles = case_when(pos872 == "C" ~ 2,
                                          pos872 == "T" ~ 0,
                                          pos872 == "t" ~ 1)) %>%
  group_by(Location) %>%
  summarise(No.blonde.alleles = sum(No.blonde.alleles),
            No.wildtype.alleles = sum(No.wildtype.alleles),
            No.individuals = n(),
            No.total.alleles = No.wildtype.alleles + No.blonde.alleles) %>%
  mutate(Freq = No.blonde.alleles / No.total.alleles)


regions <- c("Western", "Intermediate", "Intermediate", "Eastern", "Eastern",
             "Western", "Western", "Western")

data$Region <- regions

write.csv(data[c(1,7,2,3,4,5,6)], "data/processed/population_allele_table.csv", row.names = F,
          quote = F)

kable(arrange(data, desc(Region), -Freq), format = "markdown")
```

| Location | No.blonde.alleles | No.wildtype.alleles | No.individuals | No.total.alleles | Freq | Region |
| --- | --- | --- | --- | --- | --- | --- |
| SouthGeorgia | 30 | 962 | 496 | 992 | 0.0302419 | Western |
| SouthShetlands | 6 | 392 | 199 | 398 | 0.0150754 | Western |
| Bouvetoya | 2 | 932 | 467 | 934 | 0.0021413 | Western |
| Marion | 0 | 282 | 141 | 282 | 0.0000000 | Western |
| Crozet | 0 | 30 | 15 | 30 | 0.0000000 | Intermediate |
| Heard | 0 | 42 | 21 | 42 | 0.0000000 | Intermediate |
| Kerguelen | 0 | 92 | 46 | 92 | 0.0000000 | Eastern |
| Macquarie | 0 | 214 | 107 | 214 | 0.0000000 | Eastern |

Summarising the data by region:

```
regional <- data %>% group_by(Region) %>%
  summarise(No.individuals = sum(No.individuals),
            No.wildtype.alleles = sum(No.wildtype.alleles),
            No.blonde.alleles = sum(No.blonde.alleles),
            No.total.alleles = sum(No.total.alleles)) %>%
  mutate(Freq = No.blonde.alleles / No.total.alleles)

kable(arrange(regional, desc(Region)), format = "markdown")
```

| Region | No.individuals | No.wildtype.alleles | No.blonde.alleles | No.total.alleles | Freq |
| --- | --- | --- | --- | --- | --- |
| Western | 1303 | 2568 | 38 | 2606 | 0.0145817 |
| Intermediate | 36 | 72 | 0 | 72 | 0.0000000 |
| Eastern | 153 | 306 | 0 | 306 | 0.0000000 |

Total number of alleles across all populations:

```
total <- data %>% summarise(No.individuals = sum(No.individuals),
                   No.wildtype.alleles = sum(No.wildtype.alleles),
                   No.blonde.alleles = sum(No.blonde.alleles),
                   No.total.alleles = sum(No.total.alleles)) %>%
  mutate(Freq = No.blonde.alleles / No.total.alleles)

kable(total, format = "markdown")
```

| No.individuals | No.wildtype.alleles | No.blonde.alleles | No.total.alleles | Freq |
| --- | --- | --- | --- | --- |
| 1492 | 2946 | 38 | 2984 | 0.0127346 |

95% confidence intervals for blonde allele frequency:

```
# Population level: no.blonde alleles (x) over total number of alleles (n)

ci_pop <- binom.confint(data$No.blonde.alleles, 
                        data$No.total.alleles, method = "exact") %>%
  mutate(pop = data$Location,
         region = regions)

kable(arrange(ci_pop, desc(region), -mean), format = "markdown")
```

| method | x | n | mean | lower | upper | pop | region |
| --- | --- | --- | --- | --- | --- | --- | --- |
| exact | 30 | 992 | 0.0302419 | 0.0204952 | 0.0428923 | SouthGeorgia | Western |
| exact | 6 | 398 | 0.0150754 | 0.0055520 | 0.0325225 | SouthShetlands | Western |
| exact | 2 | 934 | 0.0021413 | 0.0002594 | 0.0077136 | Bouvetoya | Western |
| exact | 0 | 282 | 0.0000000 | 0.0000000 | 0.0129959 | Marion | Western |
| exact | 0 | 30 | 0.0000000 | 0.0000000 | 0.1157033 | Crozet | Intermediate |
| exact | 0 | 42 | 0.0000000 | 0.0000000 | 0.0840839 | Heard | Intermediate |
| exact | 0 | 92 | 0.0000000 | 0.0000000 | 0.0393033 | Kerguelen | Eastern |
| exact | 0 | 214 | 0.0000000 | 0.0000000 | 0.0170900 | Macquarie | Eastern |

Plot frequencies with ggplot2

```
point_size <- 4
point_alpha <- 0.4

ggplot(ci_pop, aes(fct_reorder(pop, -mean), mean)) + 
  geom_errorbar(aes(ymin=lower, ymax=upper), size = 1, width=0, col = "grey") +
  geom_point(size = point_size, fill = "grey", colour = "black", shape = 21,
             stroke = 1) + # abc_out
  theme_martin(base_family = "Lato", highlight_family = "Lato") +
 # theme_tufte(base_family = "Lato", highlight_family = "Lato") +
  theme(panel.grid.major = element_blank(),
        panel.grid.minor = element_blank(),
        plot.margin = unit(c(0.9,0.5,0.25,0.1), "cm"),
        axis.line = element_line(colour = "#cccccc"),
        axis.ticks = element_line(colour = "#cccccc"),
        axis.title.y.right = element_text(angle = 90, 
                                          margin = margin(t = 0, r = 0, b = 0, l = 15)),
        axis.text.x = element_text(angle = 60, hjust = 1)) +
  labs(x = "Population",
      y = "Frequency of S291F allele")
```

```
# Regional level: no.blonde alleles (x) over total number of alleles (n)

ci_reg <- binom.confint(regional$No.blonde.alleles, 
                        regional$No.total.alleles, method = "exact") %>%
  mutate(region = regional$Region)

kable(arrange(ci_reg, desc(region)), format = "markdown")
```

| method | x | n | mean | lower | upper | region |
| --- | --- | --- | --- | --- | --- | --- |
| exact | 38 | 2606 | 0.0145817 | 0.010339 | 0.0199601 | Western |
| exact | 0 | 72 | 0.0000000 | 0.000000 | 0.0499441 | Intermediate |
| exact | 0 | 306 | 0.0000000 | 0.000000 | 0.0119828 | Eastern |

Plot frequencies with [@ggplot2]

```
ggplot(ci_reg, aes(fct_rev(region), mean)) + 
  geom_errorbar(aes(ymin=lower, ymax=upper), size = 1, width=0, col = "grey") +
  geom_point(size = point_size, fill = "grey", colour = "black", shape = 21,
             stroke = 1) + # abc_out
  theme_martin(base_family = "Lato", highlight_family = "Lato") +
  # theme_tufte(base_family = "Lato", highlight_family = "Lato") +
  theme(panel.grid.major = element_blank(),
        panel.grid.minor = element_blank(),
        plot.margin = unit(c(0.9,0.5,0.25,0.1), "cm"),
        axis.line = element_line(colour = "#cccccc"),
        axis.ticks = element_line(colour = "#cccccc"),
        axis.title.y.right = element_text(angle = 90, 
                                          margin = margin(t = 0, r = 0, b = 0, l = 15)),
        axis.text.x = element_text(angle = 60, hjust = 1)) +
  labs(x = "Region",
       y = "Frequency of S291F allele")
```

## Fisher’s Exact Test

Run Fisher’s exact test to determine if allele frequencies are different between populations

```
# Prepare data frame

grp1 <- data %>%
  select(Location, No.wildtype.alleles, No.blonde.alleles) %>%
  slice(rep(1:(nrow(.)), each = 8))

grp2 <- data %>%
  select(Location, No.wildtype.alleles, No.blonde.alleles) %>%
    slice(rep(1:(nrow(.)), times = 8))

ft_pop <- cbind(grp1, grp2)

colnames(ft_pop) <- c("Pop1", "WT.alleles.1", "Blonde.alleles.1",
                  "Pop2", "WT.alleles.2", "Blonde.alleles.2")

ft_pop <- filter(ft_pop, Pop1 != Pop2) %>%
  filter(WT.alleles.1 > WT.alleles.2) %>%
  unite("Group", c("Pop1", "Pop2"))
```

Run Fisher’s exact test and extract p.values and odds ratios

```
ft_pop <- data.table(ft_pop, key="Group")

ft_pop <- ft_pop[, p.val := 
                   fisher.test(matrix(c(WT.alleles.1,WT.alleles.2,
                                      Blonde.alleles.1, Blonde.alleles.2), ncol=2),
                               workspace=1e9)$p.value, by=Group]

ft_pop <- ft_pop[, OR := 
                   fisher.test(matrix(c(WT.alleles.1,WT.alleles.2,
                                      Blonde.alleles.1, Blonde.alleles.2), ncol=2),
                               workspace=1e9)$estimate, by=Group]
```

Run FDR correction for multiple tests

```
# FDR correction: Benjamini Hochberg

ft_pop$adj_pval <- round(p.adjust(ft_pop$p.val, method = "BH"), 4)

kable(ft_pop, format = "markdown", digits = 20)
```

| Group | WT.alleles.1 | Blonde.alleles.1 | WT.alleles.2 | Blonde.alleles.2 | p.val | OR | adj\_pval |
| --- | --- | --- | --- | --- | --- | --- | --- |
| Bouvetoya\_Crozet | 932 | 2 | 30 | 0 | 1.0000000000000 | 0.00000000 | 1.0000 |
| Bouvetoya\_Heard | 932 | 2 | 42 | 0 | 1.0000000000000 | 0.00000000 | 1.0000 |
| Bouvetoya\_Kerguelen | 932 | 2 | 92 | 0 | 1.0000000000000 | 0.00000000 | 1.0000 |
| Bouvetoya\_Macquarie | 932 | 2 | 214 | 0 | 1.0000000000000 | 0.00000000 | 1.0000 |
| Bouvetoya\_Marion | 932 | 2 | 282 | 0 | 1.0000000000000 | 0.00000000 | 1.0000 |
| Bouvetoya\_SouthShetlands | 932 | 2 | 392 | 6 | 0.0108397043058 | 7.12074917 | 0.0759 |
| Heard\_Crozet | 42 | 0 | 30 | 0 | 1.0000000000000 | 0.00000000 | 1.0000 |
| Kerguelen\_Crozet | 92 | 0 | 30 | 0 | 1.0000000000000 | 0.00000000 | 1.0000 |
| Kerguelen\_Heard | 92 | 0 | 42 | 0 | 1.0000000000000 | 0.00000000 | 1.0000 |
| Macquarie\_Crozet | 214 | 0 | 30 | 0 | 1.0000000000000 | 0.00000000 | 1.0000 |
| Macquarie\_Heard | 214 | 0 | 42 | 0 | 1.0000000000000 | 0.00000000 | 1.0000 |
| Macquarie\_Kerguelen | 214 | 0 | 92 | 0 | 1.0000000000000 | 0.00000000 | 1.0000 |
| Marion\_Crozet | 282 | 0 | 30 | 0 | 1.0000000000000 | 0.00000000 | 1.0000 |
| Marion\_Heard | 282 | 0 | 42 | 0 | 1.0000000000000 | 0.00000000 | 1.0000 |
| Marion\_Kerguelen | 282 | 0 | 92 | 0 | 1.0000000000000 | 0.00000000 | 1.0000 |
| Marion\_Macquarie | 282 | 0 | 214 | 0 | 1.0000000000000 | 0.00000000 | 1.0000 |
| SouthGeorgia\_Bouvetoya | 962 | 30 | 932 | 2 | 0.0000002769406 | 0.06886064 | 0.0000 |
| SouthGeorgia\_Crozet | 962 | 30 | 30 | 0 | 1.0000000000000 | 0.00000000 | 1.0000 |
| SouthGeorgia\_Heard | 962 | 30 | 42 | 0 | 0.6296698131247 | 0.00000000 | 1.0000 |
| SouthGeorgia\_Kerguelen | 962 | 30 | 92 | 0 | 0.1027683981224 | 0.00000000 | 0.4111 |
| SouthGeorgia\_Macquarie | 962 | 30 | 214 | 0 | 0.0057385833607 | 0.00000000 | 0.0536 |
| SouthGeorgia\_Marion | 962 | 30 | 282 | 0 | 0.0011268332175 | 0.00000000 | 0.0158 |
| SouthGeorgia\_SouthShetlands | 962 | 30 | 392 | 6 | 0.1346596801903 | 0.49102911 | 0.4713 |
| SouthShetlands\_Crozet | 392 | 6 | 30 | 0 | 1.0000000000000 | 0.00000000 | 1.0000 |
| SouthShetlands\_Heard | 392 | 6 | 42 | 0 | 1.0000000000000 | 0.00000000 | 1.0000 |
| SouthShetlands\_Kerguelen | 392 | 6 | 92 | 0 | 0.5995305146055 | 0.00000000 | 1.0000 |
| SouthShetlands\_Macquarie | 392 | 6 | 214 | 0 | 0.0963393278337 | 0.00000000 | 0.4111 |
| SouthShetlands\_Marion | 392 | 6 | 282 | 0 | 0.0445031490179 | 0.00000000 | 0.2492 |

Run Fisher’s exact test to determine if allele frequencies are different between regions

```
#~~ Regional level

grp1_reg <- regional %>%
  select(Region, No.wildtype.alleles, No.blonde.alleles) %>%
  slice(rep(1:(nrow(.)), each = 3))

grp2_reg <- regional %>%
  select(Region, No.wildtype.alleles, No.blonde.alleles) %>%
    slice(rep(1:(nrow(.)), times = 3))

ft_reg <- cbind(grp1_reg, grp2_reg)

colnames(ft_reg) <- c("Region1", "WT.alleles.1", "Blonde.alleles.1",
                  "Region2", "WT.alleles.2", "Blonde.alleles.2")

ft_reg <- filter(ft_reg, Region1 != Region2) %>%
  filter(WT.alleles.1 > WT.alleles.2) %>%
  unite("Group", c("Region1", "Region2"))
```

Run Fisher’s exact test and extract p.values and odds ratios

```
ft_reg <- data.table(ft_reg, key="Group")
ft_reg <- ft_reg[, p.val := 
                   fisher.test(matrix(c(WT.alleles.1,WT.alleles.2,
                                      Blonde.alleles.1, Blonde.alleles.2), ncol=2),
                               workspace=1e9)$p.value, by=Group]

ft_reg <- ft_reg[, OR := 
                   fisher.test(matrix(c(WT.alleles.1,WT.alleles.2,
                                      Blonde.alleles.1, Blonde.alleles.2), ncol=2),
                               workspace=1e9)$estimate, by=Group]
```

Run FDR correction for multiple tests

```
# FDR correction: Benjamini Hochberg

ft_reg$adj_pval <- p.adjust(ft_reg$p.val, method = "BH")

kable(ft_reg, format = "markdown")
```

| Group | WT.alleles.1 | Blonde.alleles.1 | WT.alleles.2 | Blonde.alleles.2 | p.val | OR | adj\_pval |
| --- | --- | --- | --- | --- | --- | --- | --- |
| Eastern\_Intermediate | 306 | 0 | 72 | 0 | 1.0000000 | 0 | 1.0000000 |
| Western\_Eastern | 2568 | 38 | 306 | 0 | 0.0287223 | 0 | 0.0861668 |
| Western\_Intermediate | 2568 | 38 | 72 | 0 | 0.6246833 | 0 | 0.9370249 |

## GLM

```
# Read in distance data and combine with allele data

dist <- read.csv("data/raw/distance_SG.csv") %>%
  left_join(data, by = "Location")

y <- cbind(dist$No.wildtype.alleles, dist$No.blonde.alleles)

model <- glm(y ~ dist$dist_SG, binomial)
summary(model)
#> 
#> Call:
#> glm(formula = y ~ dist$dist_SG, family = binomial)
#> 
#> Deviance Residuals: 
#>      Min        1Q    Median        3Q       Max  
#> -1.29634   0.09446   0.12279   0.28019   0.77726  
#> 
#> Coefficients:
#>               Estimate Std. Error z value             Pr(>|z|)    
#> (Intercept)  3.4276467  0.1799880  19.044 < 0.0000000000000002 ***
#> dist$dist_SG 0.0008874  0.0001940   4.573            0.0000048 ***
#> ---
#> Signif. codes:  0 '***' 0.001 '**' 0.01 '*' 0.05 '.' 0.1 ' ' 1
#> 
#> (Dispersion parameter for binomial family taken to be 1)
#> 
#>     Null deviance: 47.3136  on 7  degrees of freedom
#> Residual deviance:  2.5975  on 6  degrees of freedom
#> AIC: 18.065
#> 
#> Number of Fisher Scoring iterations: 6

anova(model,test="F")
#> Warning in anova.glm(model, test = "F"): using F test with a 'binomial'
#> family is inappropriate
#> Analysis of Deviance Table
#> 
#> Model: binomial, link: logit
#> 
#> Response: y
#> 
#> Terms added sequentially (first to last)
#> 
#> 
#>              Df Deviance Resid. Df Resid. Dev      F           Pr(>F)    
#> NULL                             7     47.314                            
#> dist$dist_SG  1   44.716         6      2.598 44.716 0.00000000002278 ***
#> ---
#> Signif. codes:  0 '***' 0.001 '**' 0.01 '*' 0.05 '.' 0.1 ' ' 1
anova(model,test="Chi")
#> Analysis of Deviance Table
#> 
#> Model: binomial, link: logit
#> 
#> Response: y
#> 
#> Terms added sequentially (first to last)
#> 
#> 
#>              Df Deviance Resid. Df Resid. Dev         Pr(>Chi)    
#> NULL                             7     47.314                     
#> dist$dist_SG  1   44.716         6      2.598 0.00000000002278 ***
#> ---
#> Signif. codes:  0 '***' 0.001 '**' 0.01 '*' 0.05 '.' 0.1 ' ' 1
```

---

R version and platform.

```
sessionInfo()
#> R version 3.3.2 (2016-10-31)
#> Platform: x86_64-apple-darwin13.4.0 (64-bit)
#> Running under: macOS Sierra 10.12.6
#> 
#> locale:
#> [1] en_GB.UTF-8/en_GB.UTF-8/en_GB.UTF-8/C/en_GB.UTF-8/en_GB.UTF-8
#> 
#> attached base packages:
#> [1] stats     graphics  grDevices utils     datasets  methods   base     
#> 
#> other attached packages:
#>  [1] bindrcpp_0.2        kableExtra_0.9.0    knitr_1.20         
#>  [4] ggplot2_2.2.1       extrafont_0.17      forcats_0.2.0      
#>  [7] binom_1.1-1         data.table_1.10.4-3 tidyr_0.7.2        
#> [10] dplyr_0.7.4        
#> 
#> loaded via a namespace (and not attached):
#>  [1] Rcpp_0.12.14      highr_0.6         plyr_1.8.4       
#>  [4] bindr_0.1         tools_3.3.2       digest_0.6.13    
#>  [7] viridisLite_0.2.0 evaluate_0.10     tibble_1.3.4     
#> [10] gtable_0.2.0      pkgconfig_2.0.1   rlang_0.1.6      
#> [13] rstudioapi_0.6    yaml_2.1.14       Rttf2pt1_1.3.4   
#> [16] xml2_1.1.1        httr_1.2.1        stringr_1.2.0    
#> [19] hms_0.3           tidyselect_0.2.3  rprojroot_1.2    
#> [22] grid_3.3.2        glue_1.2.0        R6_2.2.2         
#> [25] rmarkdown_1.9     purrr_0.2.4       readr_1.1.1      
#> [28] extrafontdb_1.0   magrittr_1.5      codetools_0.2-15 
#> [31] backports_1.1.0   scales_0.5.0.9000 htmltools_0.3.6  
#> [34] assertthat_0.1    rvest_0.3.2       colorspace_1.3-2 
#> [37] labeling_0.3      stringi_1.1.2     lazyeval_0.2.1   
#> [40] munsell_0.4.3
```
